# Integration of a Droplet-Based Microfluidic System and Silicon Nanoribbon FET Sensor

**DOI:** 10.3390/mi7080134

**Published:** 2016-08-05

**Authors:** Roodabeh Afrasiabi, Lovisa M. Soderberg, Haakan N. Joensson, Per Björk, Helene Andersson Svahn, Jan Linnros

**Affiliations:** 1 Materials and Nano Physics, School of Information and Communication Technology, KTH Royal Institute of Technology, SE-164 40 Kista, Sweden; roodabeh@kth.se (R.A.); linnros@kth.se (J.L.); 2 Division of Proteomics and Nanobiotechnology, Science for Life Laboratory, KTH Royal Institute of Technology, SE-171 65 Solna, Sweden; lovisaso@kth.se (L.M.S.); helene.andersson.svahn@scilifelab.se (H.A.S.); 3 Acreo Swedish ICT AB, SE-164 40 Kista, Sweden; Per.bjork@acreo.se

**Keywords:** NanoFET, silicon nanoribbon, droplet microfluidics, pH measurement

## Abstract

We present a novel microfluidic system that integrates droplet microfluidics with a silicon nanoribbon field-effect transistor (SiNR FET), and utilize this integrated system to sense differences in pH. The device allows for selective droplet transfer to a continuous water phase, actuated by dielectrophoresis, and subsequent detection of the pH level in the retrieved droplets by SiNR FETs on an electrical sensor chip. The integrated microfluidic system demonstrates a label-free detection method for droplet microfluidics, presenting an alternative to optical fluorescence detection. In this work, we were able to differentiate between droplet trains of one pH-unit difference. The pH-based detection method in our integrated system has the potential to be utilized in the detection of biochemical reactions that induce a pH-shift in the droplets.

## 1. Introduction

Droplet microfluidics utilizes picoliter scale droplets in immiscible oil as isolated microreactors for picoliter samples e.g., single cells. The technology enables the encapsulation, parallel processing, and analysis of bioassays at a throughput of more than a thousand droplets per second [1]. Droplet microfluidics has proven advantages in the miniaturization of reactions by compartmentalization to achieve higher reaction rates and resolution [2], as well as in applications such as cell screening [3]. The most common form of detection for assays in microfluidic droplets is optical-based and relies on fluorescence [4,5,6]. Optical read-out allows for sequential individual droplet measurements on-chip but limits the assays to reactions that can be coupled to a fluorescent identifier. Other detection methods that have been explored with droplet microfluidics are based on the electrical properties of the droplets. These make use of e.g., impendence measurements [7] to detect the presence of cells in droplets, or capacitance measurements [8] where a change in the dielectric constant of the droplet content is detected.

Electrochemical sensors have shown a lot of potential as an alternative label-free detection method [9]. Potentiometric nanosensors with silicon nanoribbon field effect transistors (SiNR FETs) as the sensing elements are a category of three-terminal FET-based devices comprised of a source, a drain, and a gate structure [10]. The conductance of the channel between the source and the drain can be modulated by charging events that occur at the oxide/electrolyte interface of the transistor. The operating point of the device is set by the gate voltage and the conductance of the semiconductor channel can be turned “on”, “off”, or be modulated by the potential at the gate electrode which is connected to a reference electrode. Thus, any changes in the surface potential of the sensor can be measured and recorded in real time by the nanostructured transistors. Silicon nanowires and nanoribbons are among the most promising of such nanostructures due to their high surface area-to-volume ratio which offers high sensitivity for chemical or biological reactions that induce a change in surface charge detectable by the device [11]. Another advantage of using SiNR FET nanosensors is the sample volume needed to produce a measurable signal, which is highly reduced in FET-based nanosensors compared to other potentiometric methods [12]. The sensitivity of the SiNRs to proton concentration (pH) is described by the site-binding model for the protonation/deprotonation of the potential-determining ions at the oxide/electrolyte interface of the NRs [13]. The acidic/basic reactions of the surface hydroxyl groups (Si–OH) at the interface can be written as:

(1) $$\text{SiOH }⇄{\text{SiO}}^{-}+{\text{ H}}^{+}\text{ pH}>{\text{pH}}_{\text{pzc}}$$

(2) $${\text{SiOH}}_{2}^{+}\;⇄\text{SiOH}+{\text{ H}}^{+}\text{ pH}<{\text{pH}}_{\text{pzc}}$$

where ${\text{pH}}_{\text{pzc}}$ is the pH of the point-of-zero-charge of the gate oxide (for silicon oxide ${\text{pH}}_{\text{pzc}}=3$) [14]. In many biological reactions such as enzyme-substrate interactions, the pH of the environment is altered through acidic or basic products and SiNR FET sensors have been shown to be able to successfully detect such pH sensitive reactions [15]. The fact that substrate-enzyme concentration can be detected through pH detection eliminates the need for enzyme immobilization. The important criterion for pH detection is that consumed reactants of enzymatic reactions consist of charged species that can be quantified by SiNR FETs.

In this paper, we introduce the integration of droplet microfluidics and SiNR FETs. The droplet microfluidics enable processing of minute samples in picoliter reaction volumes which in turn can be passed to the SiNRs for label free detection that does not require the reaction to be coupled to a fluorescent molecule. SiNR FET-based detection also enables parallel measurements by the use of multiple wires, in contrast to fluorescence measurements which mainly have been restricted to sequential read-out. In this work, we demonstrate the versatility of this approach through the detection of trains of droplets with differing pH values.

**Figure 1:**
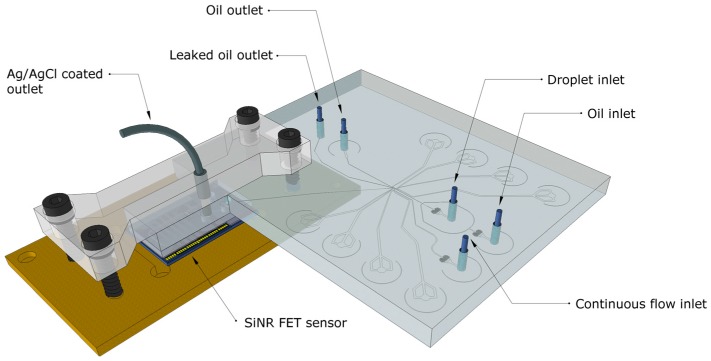
Schematic of the integrated system consisting of a microfluidic device for droplet capture integrated with a microfluidic SiNR FET sensor chip.

The integrated microfluidic device, shown in Figure 1, consists of three parts; a droplet injection module, a droplet capture component [16], an SiNR FET sensor. The integrated microfluidic device is constructed in three Polydimethylsiloxane (PDMS) layers. The two top layers form the microfluidic droplet capture device from which the liquid sample is routed to a microfluidic channel on the SiNR FET by means of vertical pathways. When a train of droplets of a different pH is captured, this induces a change in the surface potential and in the current of the nanosensor, which can then be detected electrically. The droplet capture is achieved by applying an electric field across the channel to induce dielectrophoresis, routing the combined droplet content to the continuous aqueous flow passing the SiNR FET. This droplet capture approach can also allow for sample collection such as bead extraction, where only a small amount of the emulsion is to be sampled.

Droplet microfluidics is highly dependent on optical detection and the proposed integration allows for an alternative method of droplet content detection by measuring pH changes in the continuous water phase. The pH sensitivity of FETs in combination with the droplet microfluidics can be utilized in biological applications such as enzyme–substrate interactions, cell culture applications, and flow chemistry assays. A specific example of an assay that induces a change in pH is loop-mediated isothermal amplification (LAMP) [15,17], which has previously been performed in droplets [18]. Another specific example is that of a glucose sensor [15,19,20,21,22,23] where sensing is based on the enzymatic method. The integrated system can be used to achieve oxidation of glucose in the presence of molecular oxygen in single droplets [6,16,24,25,26]. The resulting enzymatic reactions produce hydrogen peroxide, which can then be detected by the SiNR FET sensors without the need for enzyme immobilization.

## 2. Materials and Methods

### 2.1. Silicon Nanoribbon FET Fabrication

**Figure 2:**
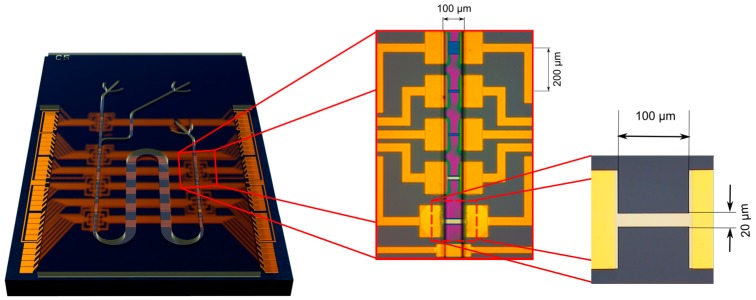
Schematics of the chip layout showing the sensor chip with all masks aligned and the structures on a single set consisting of five nanoribbons and an integrated gold pseudo-reference electrode. Optical image of the nanoribbon used in this work (100 µm × 20 µm) without the SU8 layers is shown on the right.

The devices were fabricated with a complementary metal–oxide–semiconductor (CMOS) compatible top-down process [27], using silicon-on-insulator (SOI) wafers with a buried-oxide thickness of 145 nm and a low boron doping density of 10^15^ cm^−3^. Nanoribbon structures were patterned on the device layer using UV lithography and subsequent dry-etching (device layer thickness of 30 nm and nanoribbon length and width ranging from 1 to 100 µm). In this work, nanoribbons with dimensions 100 µm × 20 µm were used. Dry thermal oxidation at 900 °C was used to form the silicon gate-oxide with a final thickness of 8 nm. The Schottky contact on the silicon pads was formed by thin film vapor deposition of 20 nm of titanium as an adhesion layer followed immediately by a 200 nm layer of gold. The chip layout is shown in Figure 2, and consists of 4 layers aligned on top of each other; the SiNR layer, the metal contact layer, the SU8 passivation layer, and the SU8 microfluidics channel layer. There are six sensor sets each with 5 nanoribbons of varying surface areas. The passivation layer insulates the chip from electrical leakage and has openings on the top of each nanoribbon for interfacing with analytes. The microfluidic channel has two inlets and one outlet and is aligned on top of the sensing areas

### 2.2. Fabrication of the On-Chip Passivation and Microchannel Systems

In order to prevent current leakage to the solution, the devices need to be electrically insulated from the solution. Using UV-lithography, a 5-µm thick SU-8 layer with open sensing areas on each separate nanoribbon was patterned on the chips as an epoxy passivation layer to protect the metal contacts from the solution and block leakage currents. Microchannels were incorporated into the sensor by patterning an additional 100-µm thick SU-8 layer on top of the passivation layer. Both layers were hard baked at 110 °C to avoid mechanical shocks and to achieve long-term stability and adhesion.

### 2.3. Microfluidic Droplet Station Setup

The droplet microfluidics experimental setup was built with an inverted microscope, Olympus IX51 (Olympus, Tokyo, Japan), and a Nemesys pump system (neMESYS, Cetoni GmbH, Korbußen, Germany) was used to actuate four syringes connected to a microfluidic chip by PEEK tubing. A high voltage amplifier (Model 623B, TREK Inc., New York, NY, USA) amplified a computer-generated square wave with a frequency of 30,000 Hz and an amplitude of 400 V. The amplifier connected to electrodes on the microfluidic chip to generate an electric field.

### 2.4. Fabrication of Droplet Microfluidic Chips

Microfluidic devices were manufactured using standard soft lithography methods [28]. Briefly, two layers of SU-8 were spin-coated on a silicon wafer and cured with UV light through a photo mask with the channel pattern, to produce a master mold. The channel patterns had a depth of 100 µm in the electrode channel and 35 µm in the microfluidic channels. The PDMS chips were fabricated by pouring PDMS base mixed with a curing agent in a 9:1 ratio on to the master mold and curing over night at 65 °C. The PDMS slab was peeled off the master mold and holes for inlets and outlets were punched with a 0.75 mm biopsy needle for water, droplet, and oil inlets, and a 2.0 mm biopsy needle for electrode channels (World Precision Instruments, Inc., Sarasota, FL, USA). The PDMS slabs were cleaned before surface activation by exposure to oxygen plasma (Cute model, Femto Science, Seoul, Korea) and bonding to a second PDMS slab. The chips were surface treated, to achieve hydrophobic channels, by injecting Aquapel (PPG Industries, Pittsburgh, PA, USA) into the channels and excess Aquapel was flushed out with pressurized nitrogen. The electrodes were fabricated by heating the devices and a low melting solder on a 95 °C hot plate or oven. The liquid solder was injected into the electrode channels and connected to an off-chip voltage source using adapters that were fixed in the channel inlets as the liquid solder solidified.

### 2.5. System Integration

The integrated device in our work is comprised of the SiNR FET sensor and the droplet-based microfluidic system. The two systems are aligned to create a complete channel system and mounted together using a removable plastic lid, which includes an Ag/AgCl coated outlet tubing used as a reference electrode. The silicon substrate of the chip is used for biasing the device through a back-gate while the integrated gold pseudo-reference electrode is kept floating.

### 2.6. Characterization of SiNR FET Sensors

Before each experiment, several I–V sweeps were taken from a single on-chip SiNR FET (In this study, 100 µm × 20 µm nanoribbon was used for all measurements) to ensure proper device performance and to check the leakage current levels. Characterization measurements were carried out using a Keithley 6485 picoammeter (Keithley, Beaverton, OR, USA) and a 2636A (Keithley, Beaverton, OR, USA) power supply. During the sweeps, the back-gate voltage (V_BG_) was applied through the silicon substrate at a constant drain voltage (V_DS_) of 1 V (source contact grounded) and the drain current (I_DS_) was recorded. Current monitoring measurements in dry condition were performed using the same system but at constant back-gate voltage.

### 2.7. Operation of the Droplet Microfluidic Device

**Figure 3:**
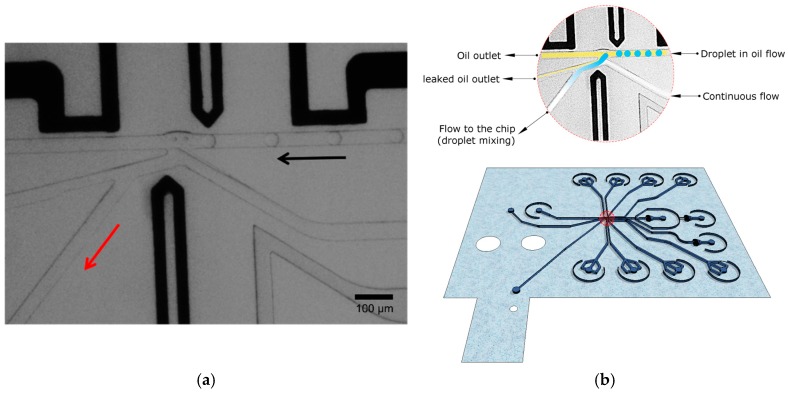
(**a**) Picture of the droplet transfer to the continuous water phase. The black arrow shows the direction of flow and the red arrow indicates the channel to the SiNR sensor; (**b**) a schematic of the droplet microfluidic chip and the droplet extraction with the oil phase in yellow, droplet content in blue, and the continuous water phase in white.

In order to test the integrated device, monodisperse aqueous droplets of 45 µm diameter were generated in an immiscible fluorinated oil (Novec HFE-7500, 3M, Zwijndrecht, Belgium) containing 0.5% EA surfactant (RainDance Technologies, Billerica, MA, USA) at the droplet injection nozzle. The flow rate in aqueous and oil phase used was 40 and 400 µL/h, respectively. The tubing connected to two syringes, mounted on syringe pumps containing buffers of different pH, and was joined with a T-junction prior to entering the chip to allow fast switching between the two solutions. The continuous water phase that leads to the SiNR FET was set to a flow rate of 70 µL/h to create a stable interface with the oil phase (Figure 3).

### 2.8. Analysis of Aqueous Droplets Using SiNR FET Sensors

For the analysis of aqueous droplets produced in oil, droplet trains with different pH values were directed to the SiNR FET-chip via a continuous aqueous stream and were detected by the SiNR FET sensors. Real-time current monitoring measurements were performed at constant V_BG_. I–V sweeps were taken before and after droplet transfer to the NRs to ensure proper performance of the device during actual detection measurements.

## 3. Results

### 3.1. Electrical Characterization of Silicon Nanoribbon FET Sensors

**Figure 4:**
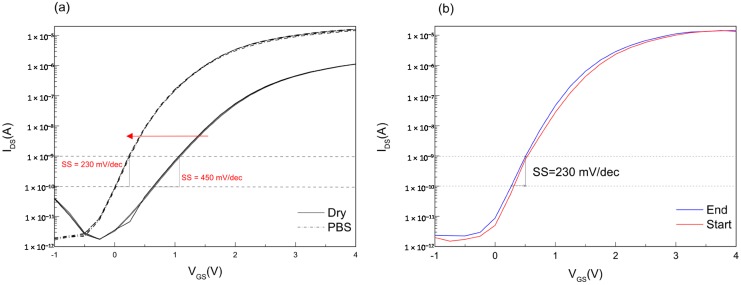
SiNR FET characterization. (**a**) IV characterization of a nanoribbon FET (100 μm × 20 μm) biased via back-gate under dry and wet conditions; (**b**) IV characterization of the same device at the start and after 5 hours of continuous buffer flow.

The electrical characteristics of the sensors in ambient conditions were obtained by applying a constant source-drain voltage and sweeping the back-gate voltage (the threshold voltage is extracted to be 2 V). Corresponding experiments were performed under wet conditions while flowing Phosphate-buffered saline (PBS) buffer solution over the chip. Figure 4a shows that the transfer characteristics of the wet sensor differs from the sensor at ambient conditions. The observed shift in IV curves is an expected device behavior caused by the charging of the oxide surface of the nanoribbons as soon as it is exposed to a buffer solution, due to protonation or deprotonation of surface hydroxyl groups.

pH sensing with SiNR FET sensors rely on shifts of either the current or the threshold voltage. It is important to account for extrinsic temporal changes in the device electrical parameters (device drift) during long measurements. For this purpose, back-gate biased IV sweeps were taken at the start and at the end of a five-hour long measurement, Figure 4b, and the voltage drift of the device observed was minimal.

### 3.2. Continuous Electric Field and pH Sensing

**Figure 5:**
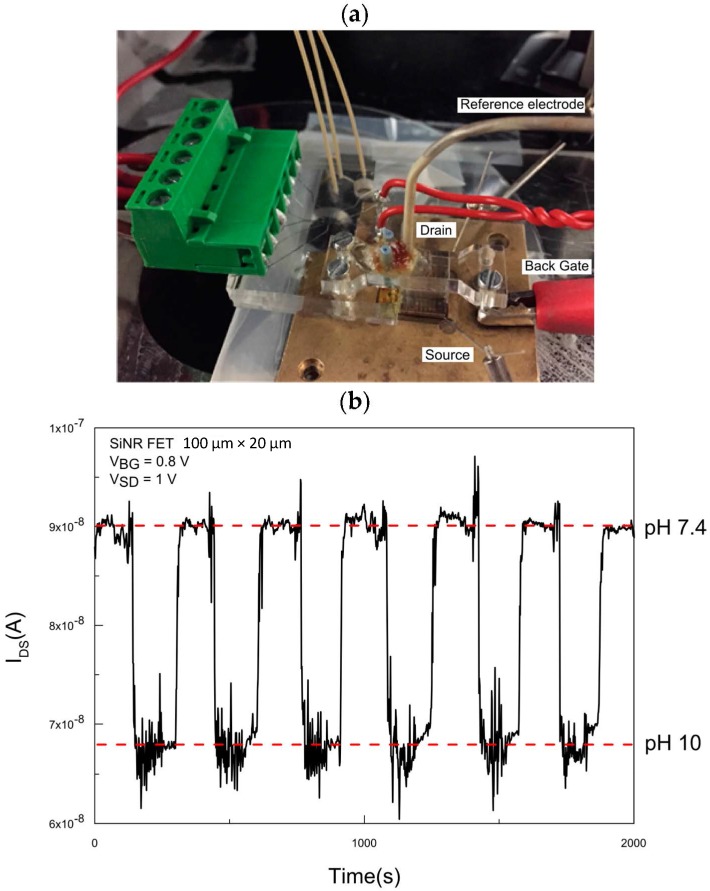
pH measurements under continuous pulse. (**a**) Set-up used for the measurements. The manual probes are used for applying source/drain voltage (V_SD_ = 1 V). The back-gate voltage (V_BG_ = 0.8 V) is applied through the substrate while the Ag/AgCl coated outlet is grounded; (**b**) Real-time current measurement showing the shift in current for the droplets with buffer solution pH 10 as soon as the pulse is turned on and the recovery to the current for PBS as soon as the pulse is turned off.

To analyze the transfer characteristics of the sensor, picoliter droplets containing an aqueous solution of a higher pH in a fluorinated oil carrier phase were transferred into a continuous aqueous stream and detected by the sensor using the newly integrated droplet-based microfluidic system (Figure 5a). When running the continuous flow (PBS pH 7.4) without droplets, as can be seen in Figure 5b, the pH level is constant and therefore the conductance remains unchanged with minimal drift. The microfluidic droplets (buffer solution pH 10) are transferred to the continuous flow as a voltage pulse is applied to the electrodes. This causes a capture of a droplet train by the continuous aqueous stream. The number of droplets included in a droplet train is determined by the time period during which the voltage pulse is applied. Here, the captured droplet contents and continuous aqueous stream mix and is subsequently directed to the sensor chip. Extraction of the contents of a train of droplets of a different pH to the continuous phase flowing across the SiNR FETs causes a shift in the pH of the continuous phase. The expected pH value of the continuous phase after droplet transfer was confirmed on the millimeter scale with a pH-meter by preparing a mixture of the buffers in a volume ratio of 9:16 ($V_{\text{droplet }}:V_{\text{continuous}}$), to simulate the used flow rates in the operation of the microfluidic device (as mentioned previously in the experimental protocol). The measured pH value was 9.4 which is close to the pH value of the stronger buffer (pH 10 here). As soon as the mixture reaches the chip, the real-time current of the SiNR FET will change in response to the addition of the droplets containing a basic buffer solution (Figure 5b). After the initial signal drop (as in the case of droplets with pH 10) and as long as the electric field is on, droplets are captured at a steady rate of 300 droplets/s and the conductance of the nanoribbon FET remains unchanged. As the electric field is turned off, no more droplets will be captured and the signal will recover to the current corresponding to the pH 7.4 level in the continuous flow.

### 3.3. Effect of the Electric Field and the Pulse-Time

In the previous section, pH sensing of picoliter droplets was performed by applying a continuous electric field until the signal from the droplets reached a steady state after which the field was turned off. One drawback of the mentioned technique is that the electric field generates substantial noise (as can be seen in Figure 5b for the current corresponding to the response from the pH 10 buffer solution), which limits the resolution and hence the signal-to-noise ratio of detection.

**Figure 6:**
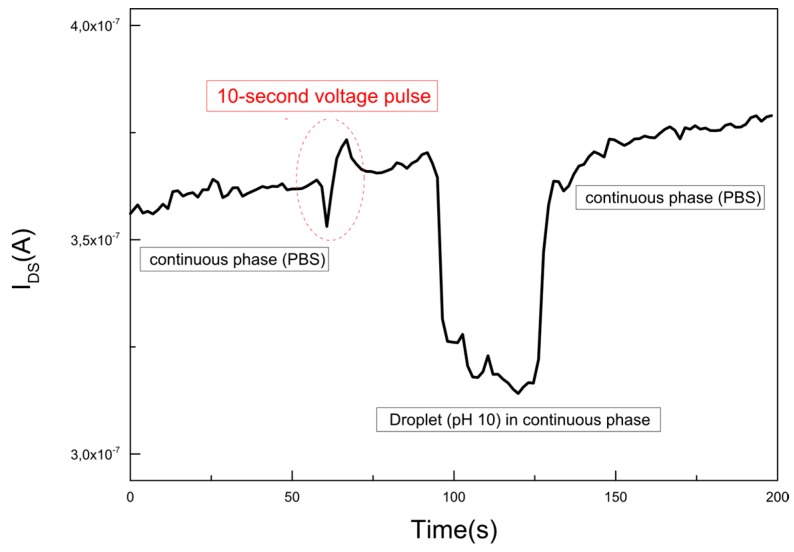
Real-time pH measurements with a 10 s pulse. The measurement starts with PBS in the continuous flow, after 50 s droplets are captured for 10 s by applying an electric field over the channel. It takes 50 s for the droplets to reach the nanoribbon at which point deprotonation of the surface hydroxyl groups results in an instant drop in the current. The signal recovers back to the current for PBS as soon as there are no droplets transferred to the continuous flow.

In order to overcome the noise, measurements were performed under different voltage pulse time period conditions. The maximum pulse time period is the time it takes for the droplet contents to reach the sensor from the transfer site, approximately 50 s for a flow rate of 125 µL/h to the SiNR FET. The conditions for minimum pulse-time require that not only a sufficient number of droplets are transferred to the chip but also for a long enough time to induce a saturated signal for the pH shift. When applying a voltage pulse shorter than the minimum required pulse-time, the current recovers before saturation of the signal. This is not desirable when comparing the signal from various pH values. A very long pulse on the other hand generates a lot of noise as discussed previously and must be avoided. Figure 6 shows the real-time pH measurements with a 10-s pulse, corresponding to approximately 3000 droplets, which was the minimum pulse-time for our system. The 10 s droplet transfer induces a longer signal drop due to Taylor Aris dispersion in the microfluidic channel leading to the SiNR FET.

### 3.4. Pulse-Controlled Droplet Transfer and pH Detection

The integrated system in this work is designed such that the captured droplets are extracted to the continuous flow before reaching the SiNR sensor. Considering that the detection is based on pH sensing, the choice of aqueous solution in the continuous flow is key to a successful detection. If a strong buffer is used in the continuous flow, then no current shift from the droplets will be detected after extraction. This can be explained by the fact that the solution in the continuous flow will buffer any pH change induced by adding the droplet content to the stream. As a result, to be able to detect the pH of the droplets, the solution in continuous flow should act merely as a dilution background with minimal effect on the pH of the droplet content. To ensure that the pH of the buffer solution (contained in the droplets) remains unaffected upon dilution, DI water was used in the continuous flow during the subsequent sensing measurements.

**Figure 7:**
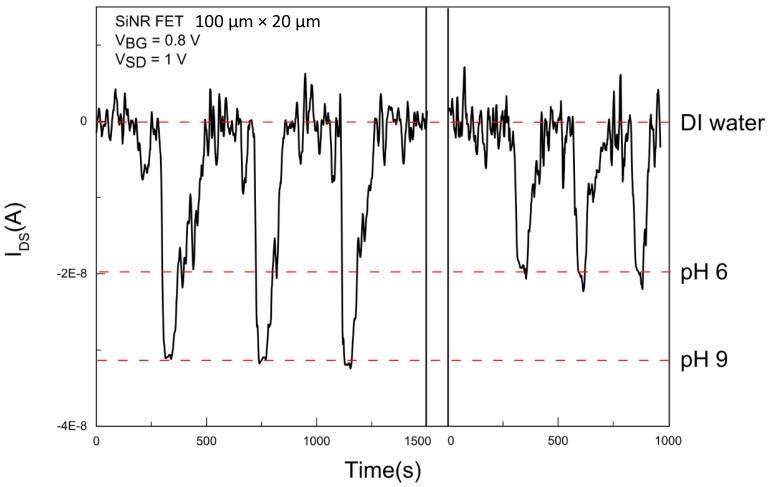
Sensing droplets with two different pH values. The background solution in the continuous flow contains DI water and following a 20-s pulse, droplets with pH 10 are transferred and mix with the continuous flow. Three consecutive pulse-controlled droplet transfers were performed with pH 9 after which the same procedure was done using pH 6 in the droplets.

Following optimization, detection experiments were performed using droplets with different pH values. For this purpose, 500 mM potassium phosphate buffers with pH of 6, 7, 8, and 9 respectively were used. As can be seen in Figure 7, the integrated system is first allowed to stabilize electrically in DI water before each droplet extraction step. After stabilization, droplet trains are captured in three 20-s intervals for each pH step. The extracted droplet trains consist of approximately 6000 droplets each due to a droplet generation of 300 droplets/s. It takes approximately 50 s for the droplet train contents to reach the SiNR FET from the start of the droplet transfer. A drop in the device current corresponds to a change in the pH of the solution over the sensor. The saturation of the pH signal is then followed by recovery and stabilization of the current back to the baseline as the wires are washed with DI water.

**Figure 8:**
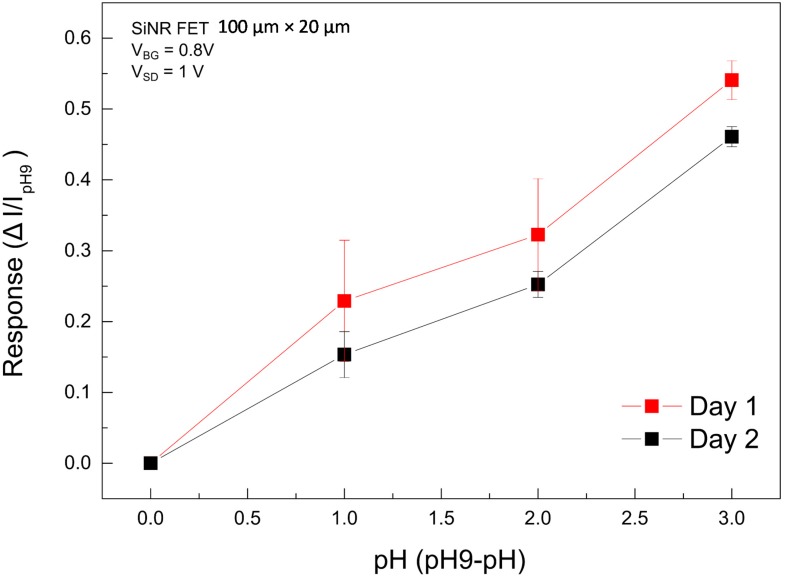
Detection of droplets with different pH values. The plot show measurements performed on the same nanoribbon and the same device. Drift in the device characteristics due to long exposure to high pH values caused a shift in the current, however the normalized response is comparable for both measurements. Data points are the mean value calculated for 3 consecutive measurements in a set pH buffer (symbols are the mean values and solid lines are guide to the eye).

Three consecutive voltage pulse-controlled measurements were taken for each pH value and in sequence of pH 6, pH 7, and pH 8. To account for the long-term current drift in the sensor and to avoid signal errors, pH 9 was used as the reference pH solution (pH 9→pH 6, pH 9→pH 7, pH 9→pH 8) to which all the pH responses were normalized. To do so, droplets with pH 9 were transferred and directed to the Nanoribbon FET during a 20-s pulse, at which time the real-time SiNR FET current drops and subsequently recovers to the DI water current at the end of the droplet extraction. The pulse-controlled measurements were repeated 3 times. This was followed by performing the same measurements for droplets with pH 6, pH 7, and pH 8, respectively, each repeated three times. To extract the response values, the DI water current was selected as the baseline and was subtracted from all pH values to account for the long term drift. The mean value and standard deviation for the current at each pH value was calculated. The pH response ($\Delta I={\text{ I}}_{\text{pH}}-I_{\text{pH}9}$) is defined as the difference between the mean values of the measured pH to pH 9. As shown in Figure 8, we were able to successfully detect and quantify the pH levels of droplet trains of each of the four pH values using this new integrated system.

## 4. Discussion

In this paper, we present the integration of two advanced technology platforms and demonstrate the label free analysis of trains of picoliter droplets with a SiNR FET sensor. With this setup we are able to distinguish between droplets containing buffers of one pH-unit difference when normalizing the obtained values to the reference buffer (pH 9). When pH measurements are repeated, small variations in the pH response to the same buffer are observed. We do observe a decrease in sensitivity of the SiNR FET after extended exposure to basic pH buffers that can be due to degradation of the thin (8 nm) gate oxide, however the pH response for the two separate measurements show the same trend. It has previously been suggested that the properties of the gate oxide can deteriorate when exposed to high acidic or basic buffer solutions and under biased conditions [29,30]. The aforementioned deviations would probably be less pronounced in most biosensing applications considering the fact that such measurements are performed in physiological media, with neutral or near neutral pH. DI-water is used as the continuous phase due to its minimal buffering capacity, to propagate the pH of the droplet train content to the SiNR FET.

The droplet capture technique can also be used to remove the surrounding oil phase for other purposes e.g., bead extraction from droplets to a continuous phase. With this technique, a small part of the droplet population can also be sampled for analysis. The design could be further improved by moving the droplet capture module closer to the SiNR FET to decrease the delay time for sensing. An additional improvement would be to add electrical shielding to the high voltage source and electrodes to suppress electrically induced noise.

Droplet microfluidics are commonly dependent on optical read-out and with this integrated device, microfluidic droplet content can be analyzed in a label-free fashion that is not coupled to a fluorescent molecule. The SiNR FET detection also allows for parallel measurements by using multiple wires that is difficult to accomplish with fluorescence. This novel system can be utilized for detection of any chemical reaction that induces a pH-shift such as loop-mediated isothermal amplification for DNA detection or hydrogen peroxide production for glucose measurements. Furthermore, biofunctionalization of the nanoribbon surface with specific biomarkers opens up more application areas for such a system as specific biosensing.
